# Recurrent oxidant treatment induces dysregulation in the brain transcriptome of Atlantic salmon (*Salmo salar*) smolts

**DOI:** 10.1016/j.toxrep.2022.06.009

**Published:** 2022-06-23

**Authors:** Danilo Carletto, Mette W. Breiland, Sigurd Hytterød, Gerrit Timmerhaus, Carlo C. Lazado

**Affiliations:** a Nofima, Norwegian Institute of Food, Fisheries and Aquaculture Research, 1433 Ås, Norway; b Department of Chemical, Biological, Pharmaceutical and Environmental Sciences, University of Messina, 98166S Agata-Messina, Italy; c Nofima, Norwegian Institute of Food, Fisheries and Aquaculture Research, 9019 Tromsø, Norway; d Norwegian Veterinary Institute, PO Box 750, Sentrum, 0106 Oslo, Norway

**Keywords:** Aquaculture, Disinfection, Fish welfare, Oxidative stress, Reactive oxygen species

## Abstract

Peracetic acid (PAA) is an organic peroxide that produces free radicals, which contribute to its potent disinfection power. At therapeutic doses, PAA is considered a mild stressor that can trigger transient local and systemic oxidative stress in fish, but the resulting consequences in the brain have yet to be identified. Therefore, we report the brain transcriptome of Atlantic salmon (*Salmo salar*) smolts that have been periodically exposed to PAA. Fish were treated three times (every 15 days) with PAA with either short (15 min) or long (30 min) exposure periods. After the third treatment, the whole brain was collected and subjected to biochemical and transcriptomic analyses. The level of reactive oxygen species in the brain was not significantly affected by recurrent PAA treatments. Microarray analysis was performed on the whole brain and revealed 205 differentially expressed genes (DEGs), regardless of the duration of the treatment. The short exposure duration had a more considerable impact on the brain transcriptome, correlating with 70% more DEGs than the long exposure. Strikingly, the brain transcriptome was characterised by the downregulation of gene expression, especially in the short exposure group, and around 82% of the identified DEGs were downregulated. Some of the highly affected genes were key molecules of the vasotocinergic and isotocinergic systems and the corticotropin-releasing factor signalling system, indicating interference of the stress axis but could also suggest an anxiolytic effect. In addition, there were alterations in genes involved in cellular metabolism and processing, signalling and trafficking, and innate immunity, which underscores the physiological changes in the brain following recurrent PAA treatment. Overall, the transcriptomic data reveal that recurrent oxidant treatment could influence brain functions, and although the magnitude was marginal, the alterations suggested neurological adaptations of fish to PAA as a potential chemical stressor. The results identify the risks of PAA, which would be valuable in drafting a framework for its empirically driven use in fish farming.

## Highlights

• Reports the brain transcriptome of Atlantic salmon smolts that have been periodically exposed to peracetic acid.
• The level of reactive oxygen species in the brain was not significantly affected by recurrent PAA treatments.
• The short exposure duration had a more considerable impact on the brain transcriptome than the long exposure.
• Majority of the differentially expressed genes were downregulated.
• The vasotocinergic and isotocinergic, and corticotropin-releasing factor signalling systems were affected.

## Graphical Abstract

**Figure:**
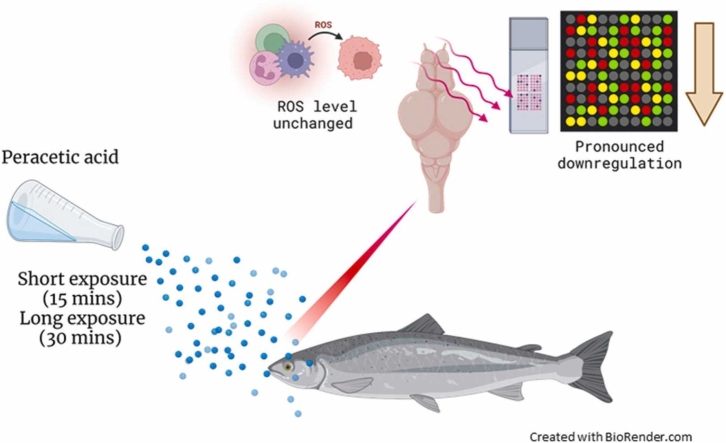

## 1 Introduction

Modern fish farming uses strategies that improve robustness through preventive measures, which are mainly achieved by enhanced biosecurity in farms, balanced and fortified nutrition, and effective vaccines [1]. However, many of these strategies are still ineffective in addressing standing bottlenecks. The only viable alternative has been to use chemotherapeutics to treat bacterial, viral, fungal, and parasitic infections. Unlike a few decades ago, when chemotherapeutics were used imprudently, modern aquaculture strives to use these treatment options cautiously, especially since resistance poses a higher risk [2].

Oxidative biocides such as chlorine, hydrogen peroxide (H_2_O_2_), and peracetic acid (PAA) are a group of oxidising agents that target many relevant fish pathogens. As oxidants, they remove electrons from susceptible chemical groups, oxidise them, and become reduced in the process [3]. PAA is one of the oxidative chemotherapeutics that has received considerable attention in the last years because of its innate features that set it apart from other commonly used therapeutics, particularly regarding safety, effectiveness, and environmental impact. Commercial PAA products are available as acidified mixtures of acetate and hydrogen peroxide, which degrade into inert and harmless residuals [4] and are potent against several fish pathogens, even at very low concentrations [5].

As an oxidising agent, PAA functions through the denaturation of protein, disruption of cell wall permeability, and oxidation of sulfhydryl and sulphur bonds in proteins, enzymes, and other metabolites [6]. The oxidative action can be a highly reversible process, and organisms have evolved many defences against the effects at lower concentrations. Nevertheless, these defence mechanisms can be exhausted at higher levels, which results in significant surface, cell wall, and intracellular damage [7], [3]. Hence, its use in aquaculture must find a balance between effectiveness against pathogens and minimising the impact on the health and welfare of host fish.

In recent years, we have progressively established the health impacts of using PAA on fish, which has revealed that salmonids (i.e., Atlantic salmon and rainbow trout) can mount strong physiological adaptive responses to PAA [8], [9], [10], [11], [12]. The series of studies on Atlantic salmon revealed that PAA application could be a mild stressor and trigger transient mucosal and systemic oxidative stress. This striking consequence is associated with the formation of radicals upon its decay, which directly interacts with the fish. It could also be due to an indirect effect of dysregulating internal redox homeostasis [4], [13]. The gills and the olfactory organ are the main organs that are sensitive to PAA in salmon. Strikingly, these mucosal organs can orchestrate a cascade of counteractive responses to the physiological threats of PAA, especially by activating the antioxidant systems.

PAA is considered a welfare-friendly antimicrobial [14]. This is exemplified by the stress responses during and following PAA treatments, indicating that fish can mobilise an adaptive response, habituate to single and repeated exposures, and demonstrate unaltered responses to a secondary stressor. However, most of our understanding of the stress physiology concerning PAA treatment in fish is focused on circulating molecules, such as the traditional indicators cortisol, glucose, and lactate [12], [14]. There have not been explorations of how the response to PAA is in the brain, a central organ of the central nervous system that regulates an array of vital processes, such as endocrine function and the stress response [15]. Environmental pollutants in the aquatic environment trigger oxidative stress and induce brain damage or dysfunction in fish [16], [17]. The brain and neurons are susceptible to reactive oxygen species (ROS), and oxidative stress has been considered a critical factor in neurotoxicity and brain injury [18]. Given the oxidative stress-inducing potential of PAA, we expect that the brain could be a target organ that influences the stress responses to the oxidant.

We hypothesise that PAA regulates brain functions, but the neurotoxicity of the therapeutic dose is low. Using microarray analysis, we report the first transcriptome of the brain of Atlantic salmon exposed to therapeutic doses of PAA. Atlantic salmon smolts were exposed periodically to PAA to simulate a husbandry scenario where fish are subjected to several rounds of oxidant treatment to prevent parasitic infection during a production cycle [10].

## 2 Materials and methods

### 2.1 Ethical use of animals for research

All procedures involving fish in this study adhered to the guidelines of the Norwegian Animal Welfare Act (Dyrevelferdsloven 2009) and Directive 2010/63/EU of the European Union (amended 2019/1010). The trial was approved by the Norwegian Food Safety Authority under FOTS ID 19321. Key personnel in the trial have FELASA C certification.

### 2.2 Recurrent exposure to peracetic acid (PAA) trial

Commercially available PAA product (Divosan Forte™ VT6) was provided by Lilleborg AS, Norway. The product is a stabilised PAA solution (15%) that is non-foaming. To ensure correct PAA dosing, the actual concentration of PAA in the product was analysed by an external laboratory (DTU Aqua, Denmark, through Dr Lars-Flemming Pedersen). The samples used were collected from an exposure trial that was reported in a sister study [10]. We confirm that no data have been duplicated in this study since a different perspective is reported regarding the large-scale *in vivo* exposure trial.

Briefly, the fish trial was performed at the Tromsø Aquaculture Station (HiT; Tromsø, Norway). Atlantic salmon smolts (approximately 80–90 g) produced at the station were distributed into nine 500-L circular tanks in a flow-through system at a density of 40 fish per tank. The system had the following parameters: a water flow rate of 6–7 L·min^−1^, salinity of 35‰, temperature of 13.0 ± 1 °C, dissolved oxygen > 90%, and saturation and photoperiod of 24 L: 0 D. A continuous feeding regime was applied (Nutra Olympic 3 mm, Skretting, Averøy, Norway). The fish were allowed to acclimatise for one week before the first PAA exposure was performed.

There were three treatment groups, and each group had three replicate tanks that were randomly distributed in the experimental hall. The two PAA treatment groups were exposed to 10 mg L^−1^ of PAA for either 15 min (short exposure) or 30 min (long exposure). Exposure was performed as follows: the water inlet was closed, and PAA was added to the water column to achieve the target concentration. Aeration was supplied to allow mixing and maintain oxygen levels > 90%. After the exposure period (15 or 30 min), the water flow was opened, and at least 90% of the water was replaced within 8–10 min. The control group was not exposed to PAA. The experimental fish were exposed to PAA every 15 days over 45 days, and there were three exposures in total. This exposure protocol mimicked an intermittent oxidant treatment in fish during a parasitic infestation.

### 2.3 Sample collection

Brain samples were collected 24 h after the last PAA exposure. Sampled fish were humanely euthanised with an overdose of benzocaine (Benzoak vet, 200 mg/mL, EuroPharma, Leknes, Norway). Five fish were collected from each tank (15 fish for each experimental group). The whole brain was dissected by making an incision on the posterior region of the skull, immediately placed in dry ice, and eventually stored at − 70 °C until analysis. Prior to ROS determination and RNA extraction, the brain samples were homogenised using a micro pestle to ensure that different regions were analysed *en masse*.

### 2.4 Determination of reactive oxygen species (ROS) in the brain

Brain lysate was prepared by suspending the tissue in sterile chilled 1X phosphate-buffered saline, ten times its volume. Samples were sonicated in ice and centrifuged at 13,000 *g* for 10 min at 4 °C. The supernatant was transferred to a new tube and immediately used for the assay. The level of ROS/RNS was determined using a commercially available kit (OxiSelect™ In Vitro ROS/RNS, CellBiolabs, Inc., USA). The assay utilises a quenched fluorogenic probe, dichlorodihydrofluorescin DiOxyQ (DCFH-DiOxyQ), a specific ROS/RNS probe. Hydrogen peroxide (H_2_O_2_) was used as the standard. All 15 fish per treatment group were used, and analyses were run in duplicate.

### 2.5 RNA isolation and microarray analysis

Automated total RNA extraction from whole brains (9 fish per treatment group) was carried out in a Biomek 4000 Benchtop Workstation using the Agencourt RNAdvance™ Tissue Total RNA Purification Kit (Beckman Coulter Inc., CA, USA). The quantity and quality of purified RNA were determined by a NanoDrop 8000 spectrophotometer (Thermo Scientific, USA). RNA quality was further assessed by an Agilent® 2100 Bioanalyzer™ RNA 6000 Nano Kit (Agilent Technology Inc., Santa Clara, CA, USA). All samples used for microarray had an RNA integrity number of 8.4 or higher. A custom-designed 15 K Atlantic salmon DNA oligonucleotide SIQ-6 microarray (Agilent Array, ICSASG_v2) was used.

RNA amplification was carried out by the One-Color Quick Amp Labelling Kit, followed by Cy3 labelling using 110 ng of RNA template per reaction. Gene expression hybridisation kits were used for the fragmentation of labelled RNA, and the arrays were hybridised for 15 h in an oven at 65 °C with a constant rotational speed of 10 rpm. Next, the arrays were successively washed with Gene Expression Wash Buffers 1 and 2 and scanned using an Agilent SureScan Microarray Scanner. Pre-processing was performed in Nofima’s bioinformatics package STARS (Salmon and Trout Annotated Reference Sequences) [19]. All reagents were purchased from Agilent Technologies.

### 2.6 Data analysis

Sigmaplot 14.0 Statistical Software (Systat Software Inc., London, UK) was used to analyse the ROS level. A student t-test was used to compare the change in ROS level in the brain and statistical significance was set at P < 0.05.

The microarray results were exported from STARS as log2 transformed expression ratios (ER) and further processed in R (version 4.0.2, https://www.r-project.org/). ERs of the treatment groups were normalised by subtracting the respective ER values of the control group. Significant differential expressed genes (DEGs) were defined by a p-value cut-off of < 0.05 (one-way ANOVA, aov() function, *stats* package) between the controls and the two treatment groups and a minimum mean ER difference of 0.5 between the highest and the lowest group. This resulted in 205 DEGs, which were represented in a heatmap (heatmap.2() function, *gplots* package, Fig. 3). Distances between genes were calculated using the Euclidean distance method, and the complete linkage algorithm calculated the dendrogram. The dendrogram was split into four clusters with distinctive expression patterns. The functional annotation terms, as they are used in STARS, were tested for significant enrichment within these clusters (fisher.test() with alternative hypothesis set to “greater” only, *stats* package). Terms with p-values < 0.05 are shown next to the heatmap with an indication in which cluster they were identified.

## 2 Results and discussion

PAA is one of the greener chemotherapeutic alternatives in aquaculture because its chemical behaviour is characterised by superior potency against diverse pathogens, rapid degradation, and inert residuals and by-products. Despite the evidence that the application of PAA could be a mild stressor for the fish, acute stress responses are not significantly affected, and robust adaptive responses are mounted, which underscores its applicability as a welfare-friendly antimicrobial agent in aquaculture [14], [10], [12]. We have made significant advancements in understanding the biology of PAA in fish, especially in regard to how it affects health and welfare, but its neurological effects remain elusive. To the best of our knowledge, this is the first report describing the brain responses at a molecular level in fish exposed to PAA. We found that salmon brains responded to recurrent PAA treatment. Moreover, the transcriptomic changes reveal that the short exposure duration had a more substantial impact on the brain than the long exposure duration.

### 2.1 Recurrent PAA exposure does not alter the ROS level in the brain

Reactive oxygen species (ROS) are reactive molecules and free radicals derived from molecular oxygen that are the vital molecular actors in oxidative stress [20]. In particular, exogenously and endogenously generated peroxides are ROS that are potent activators of cellular oxidative stress [21]. Chemotherapeutic interventions may cause oxidative stress, which is associated with cognitive impairment [22]. Brain tissue is susceptible to oxidative stress due to its limited antioxidant capacity [23]. Evidence indicates that PAA application could alter the mucosal and systemic ROS balance in fish [8], [10], [11], which provides evidence that it is a strong regulator of oxidative stress.

**Fig. 1:**
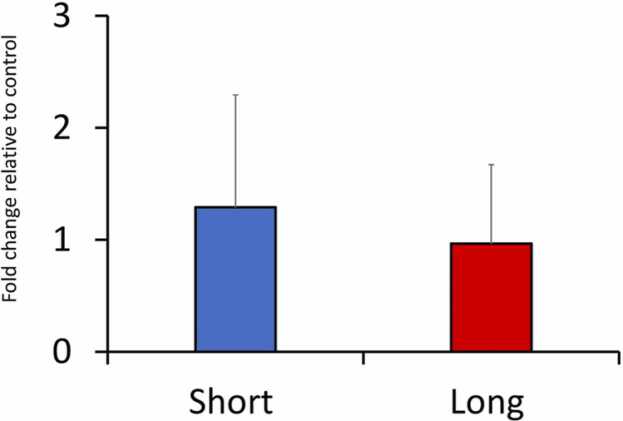
Changes in the level of ROS in the brain of Atlantic salmon smolts subjected to recurrent PAA treatment. Fold change was expressed relative to the level of ROS arbitrarily as H_2_O_2_ in the control group. There two exposure durations were short (15 min) and long (30 min). No significant change was identified. N = 15 fish.

We have shown previously that intermittent administration of PAA with either short or long exposure durations resulted in increased ROS/RNS in plasma, indicative of perturbed ROS- homeostasis [10]. In the present study, we did not find inter-treatment differences in ROS levels in the brains of salmon (Fig. 1). This was supported by the transcriptomic data showing that genes involved in oxidative stress were not considerably affected by the treatment. Hence, with the concentration and administration strategies tested, PAA administration does not trigger neurological oxidative stress via increased ROS in the brain. Xenobiotics, such as drugs and pollutants, are often observed to alter the redox balance in the brain, and this neurotoxicological effect is often used to evaluate safety [24]. Even though there were behavioural changes in response to single and recurrent exposure to PAA, which suggest neurological interference [10], [25], the present study clarifies that these may not be related to the elevation of ROS levels in the brain. This result did not correspond well with the transcriptomic changes, and hence we argue that the brain might have experienced other forms of cellular stress following PAA exposure. One of which might be in the interference of the ubiquitin-proteosome system, as discussed in the next section. The present result did not corroborate earlier evidence, especially in the ROS elevation in plasma of fish exposed intermittently to PAA [10]. This suggests that oxidative stress triggered by PAA might depend on organs, e.g. brain versus liver, and thus adding complexity to how this oxidant affects fish physiology.

Since we only have an endpoint measurement, it is also possible that we did not capture the exact time point when ROS level was elevated in the brain. The lack of samples from other timepoints limited us from further exploring this hypothesis. However, this implication is possible since earlier studies indicate that the antioxidant response to PAA in salmon is time dependent [8], [9].

### 2.2 More genes are differentially expressed in the brains of fish with short PAA exposure than long exposure

**Fig. 2:**
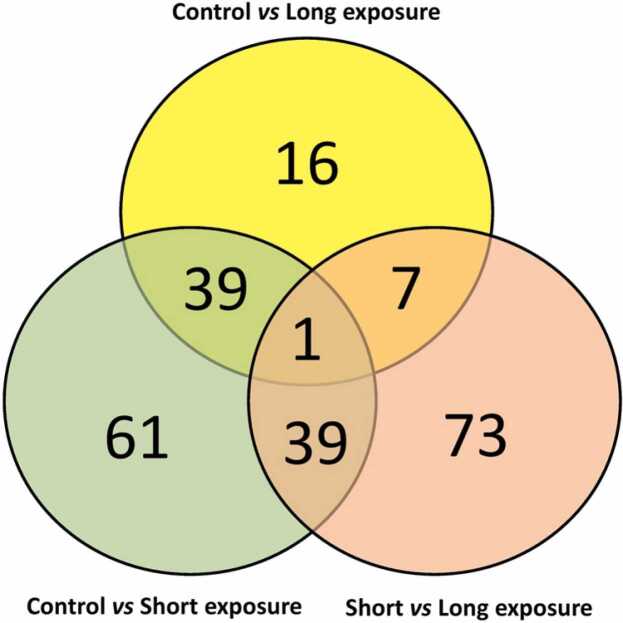
Differentially expressed genes (DEGs) in the brain of Atlantic salmon smolts subjected to recurrent PAA treatment presented as a Venn diagram showing the interactions of different group comparisons. Complete list of DEGs is supplied in Supplementary File 1.

Brain serotonergic activity has been shown to be affected by PAA [14], but the extent of its influence on brain functions in fish remains barely explored. We attempted the first molecular elucidation of the consequences of PAA on brain functions in fish. 205 differentially expressed genes (DEGs) were identified regardless of comparisons (Fig. 2, Supplementary File 1). We found 109 DEGs when short exposure was compared to the control, of which 90 were downregulated while 19 were upregulated. In contrast, only 32 DEGs were identified when long exposure was compared with the control. Around 70% (22 genes) of the DEGs identified were upregulated, demonstrating a different response profile from the short exposure versus the control. Comparing the two PAA-exposed groups, 120 DEGs were identified, and 105 of them were upregulated.

Peroxides are potentially neurotoxic and are known to alter the brain transcriptome across several organisms, including fish [23], [26]. It is apparent in the number of genes that short exposure to PAA resulted in a more substantial dysregulation in the brain than long exposure, which emphasises the regulatory influence of exposure duration on how PAA impacts brain functions. Previous reports have demonstrated that the duration of PAA exposure influences how salmonids mobilise physiological adaptive responses to PAA [11], [14]. A sister study found that long exposure instead of short exposure duration to PAA had a more considerable impact on the gills and liver transcriptome [10]. Therefore, the present data provide new insight into how the salmon brain is more sensitive to a shorter duration of PAA exposure than the organs in terms of mucosal response and hepatic metabolism of the oxidant. This further supports that responses to PAA are not only dependent on the dose and exposure duration [9], [10], but also elicit tissue-specific responses.

Interestingly, even a 15-min difference in the exposure duration could provoke a substantial contrast between the two treatments, which exemplifies the small window of the neuroregulatory function of PAA. Such a toxicological profile was also revealed in earlier studies [5]. The apparent sensitivity of the brain to the short duration could be related to the abrupt response to PAA, which was somehow abated upon more prolonged exposure.

### 2.3 Dysregulation in the brain is typified by downregulation of gene expression following recurrent PAA administration

**Fig. 3:**
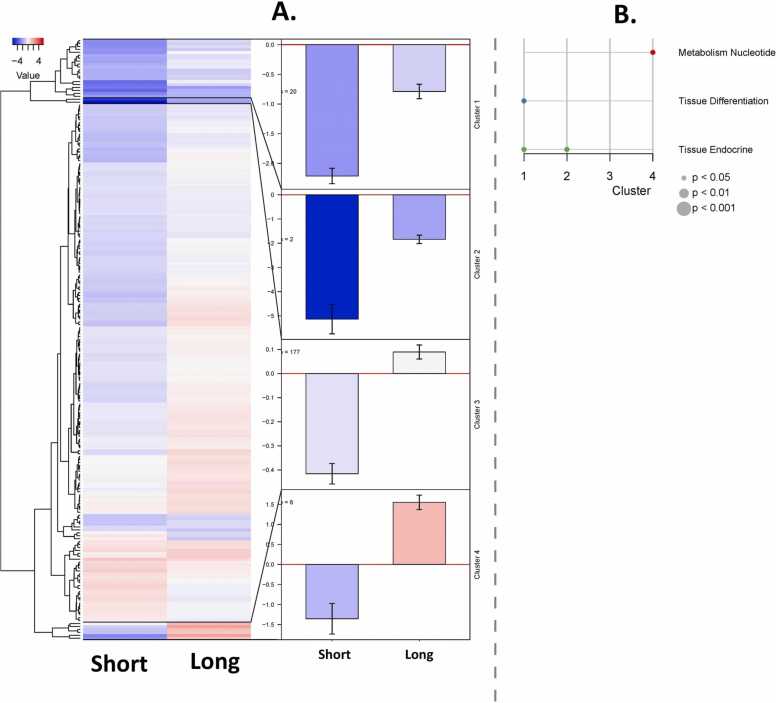
Brain transcriptome of Atlantic salmon smolts exposed to recurrent PAA treatment either short (15 min) or long (30 min) exposure duration. A) The heatmap on the left shows the down- and upregulation of DEGs in a colour gradient from blue to red. The dendrogram was split into 4 sub-clusters, and the mean values for genes within these clusters are represented in bar plots (error bars show +/- standard error of the mean) in the centre. B) Enrichment analyses of the 4 sub-clusters. The identified functional gene categories are shown along the Y-axis, and the six clusters are along the X-axis. Dots were coloured according to the categories, and the size indicates the p-value according to Fisher’s exact test.

Next, we grouped the genes according to the signature of their transcriptional profile (Fig. 3A, Table 1, Table 2, Table 3). Cluster 1 is composed of 20 genes that are typified by downregulation relative to the unexposed control group. In terms of magnitude, downregulation was more substantial with short exposure than with long exposure (Fig. 3A, Table 1). With only two genes, Cluster 2 had the lowest number. Both PAA-exposed groups showed downregulation relative to the control group. As with Cluster 1, the magnitude of the change was higher in the short-exposure group.

**Table 1:** List of differentially expressed genes in the brain from Clusters 1 and 2. Expression is given as Log2 change relative to the control group. Genes without annotated function were not included. Full list of DEGs is found in Supplementary File 1.

**Table 2:** List of differentially expressed genes in the brain from Cluster 3. Expression is given as Log2 change relative to the control group. Genes without annotated function were not included. Full list of DEGs is found in Supplementary File 1.

**Table 3:** List of differentially expressed genes in the brain from Clusters 3 (continuation) and 4. Expression is given as Log2 change relative to the control group. Genes without annotated function were not included. Full list of DEGs is found in Supplementary File 1.

Cluster 3 had the greatest number in the clustering with 177 genes (Fig. 3A, Table 1, Table 2, Table 3). This cluster is characterised by downregulation in the short-exposure group and upregulation in the long-exposure group. Lastly, Cluster 4 showed a similar tendency to Cluster 3, with downregulation observed in the short-exposure group and upregulation in the long-exposure group (Fig. 3A, Table 3). Functional enrichment of these DEGs showed no clear overall patterns (Fig. 3B). Nonetheless, we have identified three major functional groups of genes that stood out: those exhibiting downregulation were genes involved in tissue differentiation and tissue endocrine, while upregulated genes were related to nucleotide metabolism.

PAA is considered a mild stressor, and as such, exposure to it necessitates an array of stress responses in fish, which are considered an evolutionary adaptation [27]. Some of the significantly downregulated genes and exhibited considerable change were *vasotocin-neurophysin VT 1* and *isotocin-neurophysin IT 2.* These molecules are biologically active nonapeptides in teleosts produced in separate neurosecretory neurons in the hypothalamic nuclei and are involved in both osmotic and handling stress in fishes [28]. For example, in gilthead seabream (*Sparus aurata*), exposure to air for about 3 min alters the hypothalamic expression of vascotocin and isotocin precursors and receptors, which have been implicated in the activation of the stress system [29].

We have shown that PAA exposure interferes with the vasotocinergic and isotocinergic systems in the brains of salmon, which is indicative of potential inhibition of the stress axis. Repetitive exposure to PAA in salmonids has not been shown to substantially alter the ability of fish to respond to the stressor [12], [14]. In a sister study, plasma-stress parameters and behaviour following PAA exposure, either short or prolonged, did not change dramatically [10]. A number of chemical pollutants known to have neurotoxic activity in fish have been identified to disrupt the vasotocin/isotocin system [30]. This is the first report in fish showing that PAA affects this system, regardless of whether it is delivered with short or long exposure durations, and it should be cautiously considered in terms of the risk assessment concerning its use. Moreover, the inability of PAA not to trigger a strong stress response can be favourable for its use in fish as it may indicate that its stress-inducing potential is low.

Stressors elicit endocrine, autonomic, visceral, and behavioural responses from an organism, primarily coordinated by activating the corticotropin-releasing factor signalling system in the brain [31]. Two genes with key involvement in this mechanism, *corticoliberin-1-like* and *somatostatin-1A*, were downregulated following PAA exposure. This lends further support to the observation that while the general impact of PAA might marginal, there is interference to varying degrees with several molecules involved in the stress response. *Corticoliberin* (also known as *corticotropin-releasing hormone*) is the hallmark brain peptide that triggers the response to stress and mediates the stimulation of the hypothalamus-pituitary-adrenal (HPA) axis during stressful episodes. This response includes other hormonal, behavioural, autonomic, and visceral components [32]. Moreover, it has been shown to exert neuronal protection against oxidative stress [33].

*Somatostatin* comprises a relatively large class of genes that are well distributed in the brain and respond to acute stress by counteracting the various components of the stress response, such as the associated dampening of hypothalamic CRF release or its actions [31]. The transcriptomic response suggests that the tight regulation of these two molecules can be altered by recurrent PAA administration. The changes in the genes involved vasotocinergic and isotocinergic systems and the corticotropin-releasing factor signalling system in the brain suggest that PAA is an oxidant that did not induce a strong stress response, though downregulation of these molecules might indicate inhibition to respond to a potentially stressful stimulus. The long-term cost of this interference remains to be investigated, but previous studies have shown that it could be revealed in altered kinetics of the response to a secondary stressor [12].

The downregulation of vasotocin, isotocin, and corticotrophin-releasing hormone genes did not follow the classic physiological response to a potent stressor. Interestingly, such downregulation has been implicated in the anxiolytic effects of several compounds. For example, fluoxetine exerts anxiolytic effects in goldfish (*Carassius auratus*) by decreasing the isotocin mRNA levels in the brains [34], while a lowering of vasotocin mRNA abundance was observed in bluehead wrasse (*Thalassoma bifasciatum*) [35]. In another study, it has been shown that the drugs such as imipramine, fluoxetine, idazoxan, and phenelzine exhibited a similar anxiolytic effect as evidenced by decreased expression of corticotropin-releasing hormone mRNA in the paraventricular nucleus [36]. Further studies are required to validate whether PAA could reduce anxiety behaviour in fish. Corticotropin-releasing hormone correlates well with stress status. Therefore, decreasing mRNA could indicate that PAA does not trigger substantial stress system activation. This was supported by no inter-treatments differences in the levels of stress-related hormones such as cortisol, lactate and glucose between the control and the PAA-exposed groups after recurring exposures [10].

Like any other cells, brain cells respond to stress in many ways, ranging from the activation of pathways that promote survival to the elicitation of programmed cell death to eliminate damaged cells [37]. There are clear differences in how various molecules that regulate cellular activity respond to PAA administration: downregulation in the short-exposure group and upregulation in the long-exposure group, as shown by Cluster 3 in Fig. 3. Cell signalling is a complex process that is orchestrated when an organism is prompted by a stressful stimulus [38].

The homeobox genes constitute a special group of highly conserved transcription factors characterised by a common DNA binding motif [39] and tissue regeneration and repair [40]. We observed two homeobox genes that were differentially expressed in the short-exposure group: *homeobox protein DLX-1* and *distal-less homeobox gene 3b*. Their regulation might be connected to tissue repair following recurrent oxidant exposure. On the other hand, the knockdown of homeobox protein HOXB13 in HEK293 cells reduces the toxicity of oxidative stress [41]. Although this has yet to be functionally verified, the downregulation observed in the current study points to a potential role of the homeobox genes identified in resolving potential neurotoxic damage.

GTPases and related molecules play an important role in various aspects of neuronal development and functions. The Ras homolog family of guanosine triphosphate hydrolases (Rho GTPases), Ras homolog family member A (RhoA), Ras-related C3 botulinum toxin substrate 1 (Rac1), and cell division cycle 42 (Cdc42) are important regulators in somatosensory neurons, where they elicit changes in the cellular cytoskeleton. Furthermore, they are involved in diverse biological processes, including transduction of signals that contribute to fundamental cell-dynamic and survival events [42], [43]. Several genes have been identified to be differentially expressed, including *GTP-binding protein GEM*, *Ras-dva-2 small GTPase*, *Intercellular adhesion molecule 2*, *RasGEF domain family*, *member 1Ba, RAB3C, member RAS oncogene family,* and *Rho GTPase activating protein 5*. In most of these cases, downregulation was observed. Since these molecules are important for spatiotemporal fine-tuning of physiological processes, their significant regulation during oxidant exposure indicates a crucial control of cellular turnover that dictates cellular survival following intermittent exposure to a chemical stressor. GTPases have been identified to be affected in the mucosal organs of salmon exposed to PAA and were indicated to be involved in the physiological countermeasures to PAA as a stressor [8], [9]. Activation of GTPases in both mucosal and brain in salmon reveals the crucial function of these molecules following PAA exposure.

The ubiquitin-proteasome system (UPS) is the major pathway for the regulation of protein homeostasis in eukaryotic cells [44]. This process is governed by ubiquitin, a highly conserved 76-amino-acid protein that is conjugated to substrate proteins through linkage via its C-terminal glycine residue. Ubiquitin plays a vital role in degradation, DNA repair, endocytosis, and inflammation [44], [45]. Neurons rely on ubiquitin-mediated quality-control mechanisms for misfolded proteins or damaged organelles [46]. The regulation of several ubiquitin-related genes in the study, including *E3 ubiquitin-protein ligase RNF130*, *Ubiquitin-60S ribosomal protein L40* (3 transcripts), and *Ubiquitin-like protein-2,* indicates that ubiquitin-mediated processing was activated, especially for damaged proteins. We have shown earlier that ubiquitination processes were affected at the transcript level in the gills and at the protein level in skin mucus in salmon subjected to intermittent PAA treatment [10]; hence, this group of molecules play a crucial role in ensuring that damaged proteins are replaced thereby essential physiological processes at mucosal and systemic level proceed following PAA exposure.

Upregulation was explicitly exhibited in the long-exposure group, indicating that quality control via ubiquitin targets PAA-induced brain changes with a longer exposure. Mild oxidative stress has been shown to upregulate the ubiquitination system and proteasome activity in cells and tissues and transiently enhances intracellular proteolysis [47]. Although it was not convincingly established that oxidative stress was triggered locally in the brain, earlier evidence demonstrated that the PAA administration protocol in this study triggered systemic elevation of ROS [10]. The interactions among these physiological systems should be explored in future studies.

Neuronal metabolic processes in the brain ensure that nutrients and oxygen are supplied to neurons and astrocytes [48], especially when physiological demands are high, such as during exposure to a chemical stressor. Different aspects of tissue metabolism were affected following recurrent PAA treatment. For instance, the long-exposure group showed significant upregulation of genes responsible for nucleotide metabolism, such as *catechol O-methyltransferase domain-containing protein 1-like* and *High affinity cGMP-specific 3,5-cyclic phosphodiesterase*, but these were negatively and marginally affected in the short-exposure group. On the other hand, the short-exposure group showed downregulation in genes involved in calcium metabolism, such as *Calcyphosin-like protein*, *s100 calcium-binding protein*, and *Protein S100-A11*, but the opposite was observed in the long-exposure group.

Dysregulation of neuronal intracellular Ca2 + homeostasis can play a crucial role in many neurotoxic effects, including impaired brain functions and behaviour [49]. It could be possible that recurrent PAA treatment alters the Ca2 + balance in the brain, which increases the risk of neurotoxicity of PAA with a shorter exposure. It has been reported that the metabolic function of calcium is crucial during oxidative stress through activation of the membrane permeability transition, release of cytochrome c, and respiratory inhibition, among others [50]. We believe that this is also involved in salmon exposed to PAA.

Proteolytical processing of membrane-bound molecules is a fundamental mechanism for the degradation of these proteins and controlling cell-to-cell communication [51]. Gene encoding for proteases was also represented in the set of DEGs, including *serine (or cysteine) proteinase inhibitor clade E, nexin, plasminogen activator inhibitor type 1-like*, *plasma protease C1 inhibitor-like*, *Aminopeptidase N*, *trypsin inhibitor ClTI-1-like,* and *Kunitz-type protease inhibitor 2 precursor*. In most cases, downregulation was observed in the short-exposure group, while upregulation was demonstrated in the long-exposure group. This differing response suggests that proteasomal and lysosomal proteolytic pathways that continually maintain protein turnover are inhibited by short exposure duration. The relevance of such inhibition to the neurological risk of PAA remains to be functionally elucidated, but this observation warrants consideration in assessing the health risk of PAA to salmon.

Fish have an established neuroimmune interaction [52], but this interplay is not often explored in the context of chemotherapeutics administration. Studies have demonstrated that PAA is a potent modulator of immune functions in salmon, particularly at the mucosal surfaces [8], [9]. We have identified some immune genes that are affected by PAA treatment, including those involved in T cells (*modified T cell receptor alpha*), cytokines (*interferon gamma receptor 1*, *interleukin-16*), and the complement system (*Complement C3*, *Mannan binding lectin serine proteases*, *C1q-like adipose protein*, *complement C2-like*, *Complement factor Bf-1*). Microglial cells are the main innate immune cells of the complex cellular structure of the brain and they respond quickly to pathogens, stress, and injury by activating a cascade of pro-inflammatory responses [53]. The complement system is crucial for microglial cells [54]. It consists of over 30 independent proteins and provides rapid recognition and response to danger to the host [55]. Aside from their key roles in defence, complement proteins in the brain exert non-inflammatory functions in regulating structural plasticity and functional homeostasis of synapses [54]. Their considerable regulation of several complement genes following recurrent exposure to PAA is perhaps related to ensuring brain homeostasis, which is crucial for the adaptive response to the oxidant.

In summary, this study has presented the first brain transcriptome data from fish subjected to PAA treatment. Overall, the transcriptomic changes indicate that recurrent exposure to PAA alters brain functions, but the magnitude seems marginal given the number of differentially expressed genes compared with previous transcriptomics studies on salmon smolts exposed to PAA [8], [9], [10]. Although it was not quite clear whether PAA triggered oxidative stress in the brain, genes involved in stress responses were affected, especially those involved in the hypothalamic-pituitary-interrenal (HPI) axis.

Differentially expressed genes indicate that the short exposure had a substantially greater impact on the brain than the long exposure. The results offer new insight that even a 15-min window of exposure has consequential impacts on the brain functions. These transcriptomic alterations present another perspective on how PAA could produce interference and possibly pose a threat if treatment protocols are not executed properly. This in spite of PAA generally being considered as a welfare-friendly antimicrobial for fish [11], [14]. Genes that were found to be responsive to PAA could be used as potential markers for physiological impacts of the treatment and should be verified in further studies. In addition, these results should be valuable in guiding the evidence-driven use of PAA in aquaculture, particularly as a chemotherapeutic.

## CRediT authorship contribution statement

C.C.L. conceived the research idea; C.C.L., M.W.B. and S.H. designed the trial; C.C.L., M.W.B., D.S., and S.H. performed the fish trial; C.C.L., M.W.B., and S.H. collected the samples; D.C. performed the lab analyses; C.C.L. supervised D.C; C.C.L., G.T., and D.C. handled and processed the data; D.C. and C.C.L. interpreted the data; C.C.L. and D.C. wrote the first draft of the manuscript. All authors contributed to the writing and review of the final version of the manuscript. All authors contributed to the writing and review of the final version of the manuscript.

## Declaration of Competing Interest

No conflict of interest.

## References

[1] Assefa A., Abunna F. (2018). Maintenance of fish health in aquaculture: review of epidemiological approaches for prevention and control of infectious disease of fish. Vet. Med. Int..

[2] Schar D., Zhao C., Wang Y., Larsson D.G.J., Gilbert M., Van Boeckel T.P. (2021). Twenty-year trends in antimicrobial resistance from aquaculture and fisheries in Asia. Nat. Commun..

[3] Finnegan M., Linley E., Denyer S.P., McDonnell G., Simons C., Maillard J.-Y. (2010). Mode of action of hydrogen peroxide and other oxidizing agents: differences between liquid and gas forms. J. Antimicrob. Chemother..

[4] Pedersen L.-F., Lazado C.C. (2020). Decay of peracetic acid in seawater and implications for its chemotherapeutic potential in aquaculture. Aquac. Environ. Interact..

[5] Straus D.L., Meinelt T., Liu D., Pedersen L.-F. (2018). Toxicity of peracetic acid to fish: variation among species and impact of water chemistry. J. World Aquac. Soc..

[6] Freeman D.E., Auer J.A., Auer J.A., Stick J.A. (2012). Equine Surgery.

[7] Acosta F., Montero D., Izquierdo M., Galindo-Villegas J. (2021). High-level biocidal products effectively eradicate pathogenic γ-proteobacteria biofilms from aquaculture facilities. Aquaculture.

[8] Lazado C.C., Pedersen L.-F., Kirste K.H., Soleng M., Breiland M.W., Timmerhaus G. (2020). Oxidant-induced modifications in the mucosal transcriptome and circulating metabolome of Atlantic salmon. Aquat. Toxicol..

[9] Lazado C.C., Sveen L.R., Soleng M., Pedersen L.-F., Timmerhaus G. (2021). Crowding reshapes the mucosal but not the systemic response repertoires of Atlantic salmon to peracetic acid. Aquaculture.

[10] Lazado C.C., Timmerhaus G., Breiland M.W., Pittman K., Hytterød S. (2021). Multiomics provide insights into the key molecules and pathways involved in the physiological adaptation of Atlantic salmon (*Salmo salar*) to chemotherapeutic-induced oxidative stress. Antioxidants.

[11] Liu D., Lazado C.C., Pedersen L.-F., Straus D.L., Meinelt T. (2020). Antioxidative, histological and immunological responses of rainbow trout after periodic and continuous exposures to a peracetic acid-based disinfectant. Aquaculture.

[12] Osório J., Stiller K.T., Reiten B.-K., Kolarevic J., Johansen L.-H., Afonso F., Lazado C.C. (2022). Intermittent administration of peracetic acid is a mild environmental stressor that elicits mucosal and systemic adaptive responses from Atlantic salmon post-smolts. BMC. Zoology.

[13] Rokhina E.V., Makarova K., Golovina E.A., Van As H., Virkutyte J. (2010). Free radical reaction pathway, thermochemistry of peracetic acid homolysis, and its application for phenol degradation: spectroscopic study and quantum chemistry calculations. Environ. Sci. Technol..

[14] Gesto M., Liu D., Pedersen L.-F., Meinelt T., Straus D.L., Jokumsen A. (2018). Confirmation that pulse and continuous peracetic acid administration does not disrupt the acute stress response in rainbow trout. Aquaculture.

[15] Cerdá‐Reverter J.M., Canosa L.F. (2009). Fish Physiology.

[16] Baldissera M.D., Souza C.F., da Silva A.S., Henn A.S., Flores E.M.M., Baldisserotto B. (2020). Diphenyl diselenide dietary supplementation alleviates behavior impairment and brain damage in grass carp (Ctenopharyngodon idella) exposed to methylmercury chloride. Comp. Biochem. Physiol. Part C Toxicol. Pharmacol..

[17] Shaw P., Mondal P., Bandyopadhyay A., Chattopadhyay A. (2020). Environmentally relevant concentration of chromium induces nuclear deformities in erythrocytes and alters the expression of stress-responsive and apoptotic genes in brain of adult zebrafish. Sci. Total Environ..

[18] Pei D.S., Jia P.P., Luo J.J., Liu W., Strauss P.R. (2019). AP endonuclease 1 (Apex1) influences brain development linking oxidative stress and DNA repair. Cell Death Dis..

[19] Krasnov A., Timmerhaus G., Afanasyev S., Jørgensen S.M. (2011). Development and assessment of oligonucleotide microarrays for Atlantic salmon (*Salmo salar* L.). Comp. Biochem. Physiol. Part D Genom. Proteom..

[20] Lushchak V.I. (2014). Free radicals, reactive oxygen species, oxidative stress and its classification. Chem. Biol. Interact..

[21] Ransy C., Vaz C., Lombès A., Bouillaud F. (2020). Use of H(2)O(2) to cause oxidative stress, the catalase issue. Int. J. Mol. Sci..

[22] Gaman A.M., Uzoni A., Popa-Wagner A., Andrei A., Petcu E.-B. (2016). The role of oxidative stress in etiopathogenesis of chemotherapy induced cognitive impairment (CICI)-“Chemobrain”. Aging Dis..

[23] Salim S. (2017). Oxidative stress and the central nervous system. J. Pharmacol. Exp. Ther..

[24] Cardoso O., Puga S., Brandão F., Canário J., O’Driscoll N.J., Santos M.A., Pacheco M., Pereira P. (2017). Oxidative stress profiles in brain point out a higher susceptibility of fish to waterborne divalent mercury compared to dietary organic mercury. Mar. Pollut. Bull..

[25] Mota V.C., Eggen M.L., Lazado C.C. (2022). Acute dose-response exposure of a peracetic acid-based disinfectant to Atlantic salmon parr reared in recirculating aquaculture systems. Aquaculture.

[26] Jia R., Du J., Cao L., Feng W., He Q., Xu P., Yin G. (2021). Application of transcriptome analysis to understand the adverse effects of hydrogen peroxide exposure on brain function in common carp (Cyprinus carpio). Environ. Pollut..

[27] Petitjean Q., Jean S., Gandar A., Côte J., Laffaille P., Jacquin L. (2019). Stress responses in fish: from molecular to evolutionary processes. Sci. Total Environ..

[28] Sokołowska E., Gozdowska M., Kulczykowska E. (2020). Nonapeptides arginine vasotocin and isotocin in fishes: advantage of bioactive molecules measurement. Front. Mar. Sci..

[29] Skrzynska A.K., Maiorano E., Bastaroli M., Naderi F., Míguez J.M., Martínez-Rodríguez G., Mancera J.M., Martos-Sitcha J.A. (2018). Impact of air exposure on vasotocinergic and isotocinergic systems in Gilthead Sea Bream (Sparus aurata): new insights on fish stress response. Front. Physiol..

[30] Kalamarz-Kubiak H. (2021). Endocrine-disrupting compounds in fish physiology, with emphasis on their effects on the arginine vasotocin/isotocin system. Endocr. Metab. Immune Disord. Drug Targets.

[31] Stengel A., Rivier J., Taché Y. (2013). Modulation of the adaptive response to stress by brain activation of selective somatostatin receptor subtypes. Peptides.

[32] Stengel A., Taché Y.F. (2017). Activation of brain somatostatin signaling suppresses CRF receptor-mediated stress response. Front. Neurosci..

[33] Lezoualc’h F., Engert S., Berning B., Behl C. (2000). Corticotropin-releasing hormone-mediated neuroprotection against oxidative stress is associated with the increased release of non-amyloidogenic amyloid beta precursor protein and with the suppression of nuclear factor-kappaB. Mol. Endocrinol..

[34] Mennigen J.A., Martyniuk C.J., Crump K., Xiong H., Zhao E., Popesku J., Anisman H., Cossins A.R., Xia X., Trudeau V.L. (2008). Effects of fluoxetine on the reproductive axis of female goldfish (Carassius auratus). Physiol. Genom..

[35] Semsar K., Perreault H.A., Godwin J. (2004). Fluoxetine-treated male wrasses exhibit low AVT expression. Brain Res..

[36] Brady L.S., Gold P.W., Herkenham M., Lynn A.B., Whitfield H.J. (1992). The antidepressants fluoxetine, idazoxan and phenelzine alter corticotropin-releasing hormone and tyrosine hydroxylase mRNA levels in rat brain: therapeutic implications. Brain Res..

[37] Fulda S., Gorman A.M., Hori O., Samali A. (2010). Cellular stress responses: cell survival and cell death. Int. J. Cell Biol..

[38] Hotamisligil G.S., Davis R.J. (2016). Cell signaling and stress responses. Cold Spring Harb. Perspect. Biol..

[39] Dankel S.N., Fadnes D.J., Stavrum A.-K., Stansberg C., Holdhus R., Hoang T., Veum V.L., Christensen B.J., Våge V., Sagen J.V., Steen V.M., Mellgren G. (2010). Switch from stress response to homeobox transcription factors in adipose tissue after profound fat loss. PLoS One.

[40] Wang K.C., Helms J.A., Chang H.Y. (2009). Regeneration, repair and remembering identity: the three Rs of Hox gene expression. Trends Cell Biol..

[41] Nakano R., Takahashi T., Naganuma A., Hwang G.W. (2013). Knockdown of the gene for homeobox protein HOXB13 reduces toxicity of oxidative-stress inducers in HEK293 cells. J. Toxicol. Sci..

[42] Boueid M.-J., Mikdache A., Lesport E., Degerny C., Tawk M. (2020). Rho GTPases signaling in zebrafish development and disease. Cells.

[43] Kalpachidou T., Spiecker L., Kress M., Quarta S. (2019). Rho GTPases in the physiology and pathophysiology of peripheral sensory neurons. Cells.

[44] Shruthi K., Reddy S.S., Chitra P.S., Reddy G.B. (2019). Ubiquitin-proteasome system and ER stress in the brain of diabetic rats. J. Cell Biochem.

[45] McClellan A.J., Laugesen S.H., Ellgaard L. (2019). Cellular functions and molecular mechanisms of non-lysine ubiquitination. Open Biol..

[46] Schmidt M.F., Gan Z.Y., Komander D., Dewson G. (2021). Ubiquitin signalling in neurodegeneration: mechanisms and therapeutic opportunities. Cell Death Differ..

[47] Shang F., Taylor A. (2011). Ubiquitin-proteasome pathway and cellular responses to oxidative stress. Free Radic. Biol. Med..

[48] Zoccoli G., Silvani A., Franzini C. (2017).

[49] Dusza H.M., Cenijn P.H., Kamstra J.H., Westerink R.H.S., Leonards P.E.G., Hamers T. (2018). Effects of environmental pollutants on calcium release and uptake by rat cortical microsomes. Neurotoxicology.

[50] Starkov A.A., Chinopoulos C., Fiskum G. (2004). Mitochondrial calcium and oxidative stress as mediators of ischemic brain injury. Cell Calcium.

[51] Saftig P., Bovolenta P. (2015). Proteases at work: cues for understanding neural development and degeneration. Front. Mol. Neurosci..

[52] Salinas I., Sepahi A., Kraus A., Das P. (2019). Rainbow trout as a model for the study of neuroimmune interactions in the nasal mucosa. J. Immunol..

[53] Rivest S. (2009). Regulation of innate immune responses in the brain. Nat. Rev. Immunol..

[54] Stevens B., Johnson M.B. (2021). The complement cascade repurposed in the brain. Nat. Rev. Immunol..

[55] Fonseca M.I., Chu S.-H., Hernandez M.X., Fang M.J., Modarresi L., Selvan P., MacGregor G.R., Tenner A.J. (2017). Cell-specific deletion of C1qa identifies microglia as the dominant source of C1q in mouse brain. J. Neuroinflamm..

